# Endocytosis as a mode to regulate functional expression of two-pore domain potassium (K_2P_) channels

**DOI:** 10.1007/s00424-014-1641-9

**Published:** 2014-11-22

**Authors:** Ita O’Kelly

**Affiliations:** Human Development and Health, Centre for Human Development, Stem Cells and Regeneration, Faculty of Medicine, University of Southampton, Southampton, SO16 6YD UK

**Keywords:** K_2P_ channel, Endocytosis, Recycling, Clathrin, TWIK, TREK, TASK, TRESK, Potassium channel

## Abstract

Two-pore domain potassium (K_2P_) channels are implicated in an array of physiological and pathophysiological roles. As a result of their biophysical properties, these channels produce a background leak K^+^ current which has a direct effect on cellular membrane potential and activity. The regulation of potassium leak from cells through K_2P_ channels is of critical importance to cell function, development and survival. Controlling the cell surface expression of these channels is one mode to regulate their function and is achieved through a balance between regulated channel delivery to and retrieval from the cell surface. Here, we explore the modes of retrieval of K_2P_ channels from the plasma membrane and observe that K_2P_ channels are endocytosed in both a clathrin-mediated and clathrin-independent manner. K_2P_ channels use a variety of pathways and show altered internalisation and sorting in response to external cues. These pathways working in concert, equip the cell with a range of approaches to maintain steady state levels of channels and to respond rapidly should changes in channel density be required.

## Introduction

Cellular endocytosis was classically viewed as a mechanism for protein internalisation and destruction. The identification of multiple endocytic pathways with defined cellular destinations helped recognise endocytosis as not only a means of reducing the expression of membrane proteins but also a mechanism to enable their rapid recycling and redistribution [13, 28, 78]. Endocytosis is now understood to be central to the fine control of cell surface expression and function of many membrane proteins.

Expression of two-pore domain potassium (K_2P_) channels on the cell surface is regulated at the transcriptional and post-transcriptional levels, via controlled biogenesis, sorting and trafficking [32, 65, 73]. These channels show constitutive activity once inserted into the plasma membrane and their surface expression directly impacts cell membrane potential by supporting K^+^ leak from cells [43, 65, 69]. Consequently, changes to channel surface density will result in changes in K^+^ leak, membrane depolarisation and hence, cellular function and excitation. Furthermore, as the 15 K_2P_ family members show widespread tissue distribution and, as reviewed by others within this issue, are proposed to play roles in cellular mechanisms as diverse as chemoreception, adrenal development, cardiac function, pain, sleep and anaesthesia, the control of their surface density has the potential to impact an array of cellular physiological and pathophysiological processes [16, 46, 47, 90]. Our understanding of the mechanisms which regulate delivery of members of the K_2P_ family to the cell surface has been elucidated over the last decade and is the focus of Renigunta et al. review (in this issue). Clearly, the recovery of K_2P_ channels from the plasma membrane is of equal importance and a molecular understanding of the interplay between various endocytic pathways and environmental triggers is vital to our understanding of their roles in cell regulation. To date, we have limited knowledge of the pathways used by many of the K_2P_ channels. This article will provide an overview of our current understanding of the predominant endocytic pathways utilised by ion channels and present recent evidence of endocytic pathway usage by members of the K_2P_ family, including K_2P_1.1 (TWIK1), K_2P_2.1 (TREK1), K_2P_3.1 (TASK1) K_2P_9.1 (TASK3) and K_2P_18.1 (TRESK). Finally, using known criteria for recruitment of cargo into specific endocytic pathways, we speculate on likely modes of endocytosis of the remaining K_2P_ family members.

## Ion channel endocytosis

**Fig. 1:**
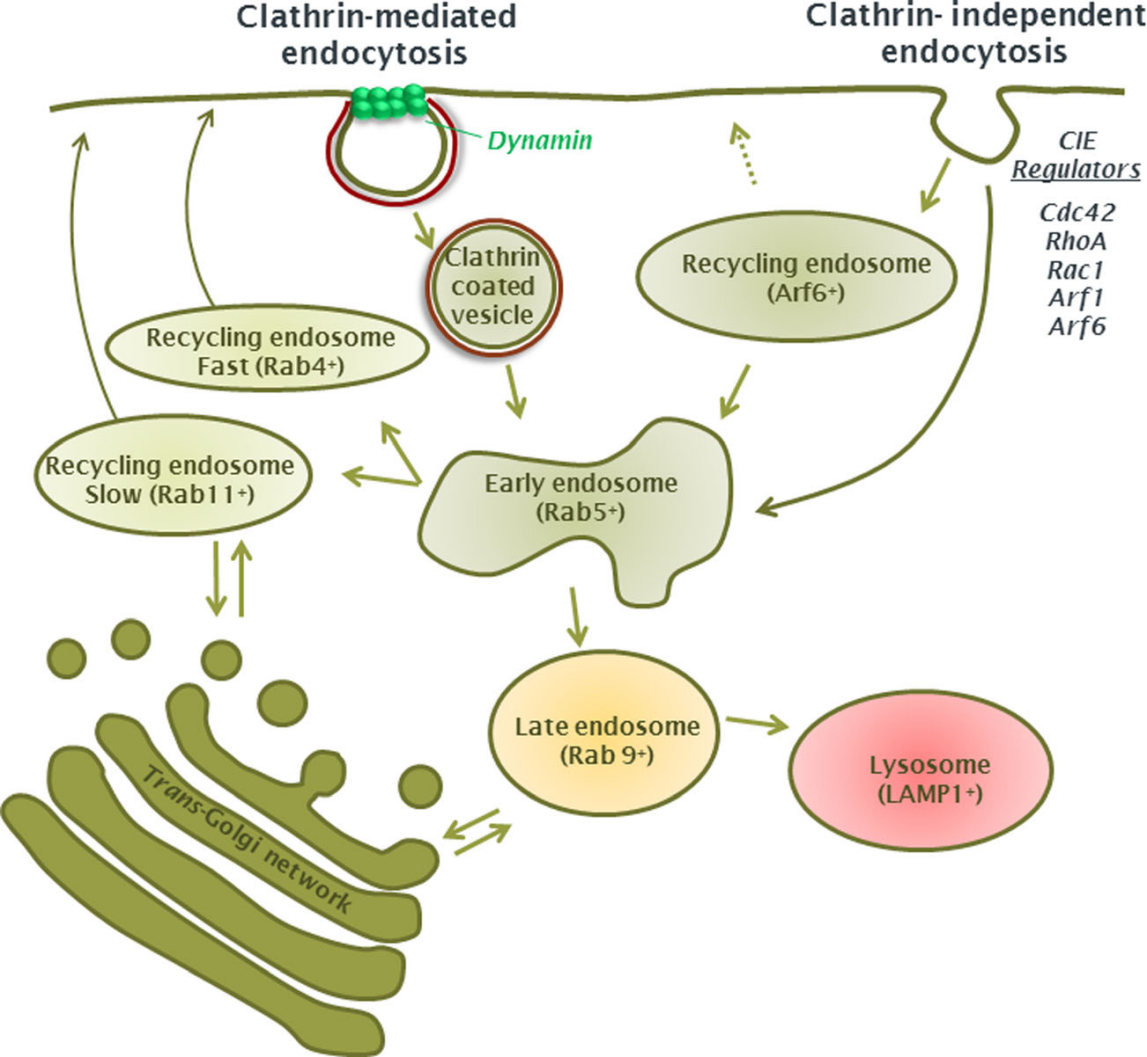
Ion channel endocytic pathways. Clathrin-mediated and clathrin-independent sorting pathways utilised by ion channels. Channels can be internalised by scission of clathrin-coated pits by action of dynamin to produce clathrin-coated vesicles which can be sorted to endosomes, lysosomes and trans-Golgi network. Cargo internalised through clathrin-independent pathways can also be sorted to the same destinations following their shuffling to the early endosomes (Rab5^+^). Alternatively, cargo of the clathrin-independent pathway can enter ARF6^+^ recycling endosomes and return directly to the cell surface or transit to the early endosomes and into the endocytic pathway (recycling or late endosomes) from there. Prominent Rab proteins in each of the compartments are included. *Arrows* indicate possible direction of sorting between compartments

Cells use various mechanisms to internalise plasma membrane proteins (Fig. 1), and many proteins are capable of being recruited to different endocytic pathways in response to environmental triggers or as a result of constitutive or stimulated endocytosis [17, 20, 110]. The primary congregation point for most internalised proteins is the Rab5 (Ras-related GTPase 5) positive early endosome [11, 64, 114]. From here, proteins can either transit to recycling endosomes and back to the plasma membrane or are sorted to late endosomes and finally to the lysosome for degradation [11, 12, 93]. Similarly, at the trans-Golgi network (TGN), proteins can shuttle to the plasma membrane or be diverted to endosomes [12, 54]. An ARF6 (a member of the family of ADP-ribosylation factor) positive compartment operates as an alternative route to direct delivery of cargo to the Rab5 positive early endosomes [15, 59, 95, 96]. Proteins targeted to the ARF6 positive compartment can be recycled directly back to the cell surface or pass from there to early endosomes. On the whole, the endocytic routes and subsequent trafficking pathways (degradation versus recycling) are specified by structural features or sorting motifs within the cytoplasmic domains of cargo proteins [11, 114]. These endocytic signals enable binding partners to target the protein into specific pathways. While the molecular details of these processes are still emerging, recognised motifs and modifications have been defined.

## Internalisation and intracellular sorting signals

The most extensively studied endocytic pathway is clathrin-mediated endocytosis (CME); however, a range of additional clathrin-independent endocytic pathways operate within different cell types and are proposed to account for a significant proportion of protein endocytosis [22, 28, 53, 75, 87]. Adaptor proteins (predominantly but not exclusively AP 1–4) together with clathrin-associated sorting proteins (CLASPs) recruit cargo into clathrin-coated vesicles of the CME pathway [62, 87, 92, 113]. Recruitment relies on recognition of sorting motifs predominantly within the cytosolic termini of cargo protein. A tyrosine motif (YXXϕ; using single amino acid code X represents a variable residue and ϕ a hydrophobic residue) and a di-leucine [DE]XXXL [LI] motif in channel c-termini are recognised by clathrin adaptor protein AP-2 (μ and α-δ2 subunits, respectively) [11]. AP-2 binding facilitates recruitment to the clathrin bud and results in channel endocytosis and lysosomal targeting. AP-1 and AP-3 also recognise these motifs but play roles in bidirectional transport between TGN and endosomes and sorting to lysosome (for AP-3 recruited cargo) [104, 109]. A similar motif, DXXLL or acidic cluster/di-leucine motif, is recognised by another member of the ARF family (ARF1) which is localised to the TGN and endosomes and regulates membrane recruitment of AP1 and AP3 [30, 81]. These motifs are thought not to be involved in cargo internalisation or recycling but likely enable sorting of transmembrane proteins from the TGN to endosomes.

Cargo bearing an alternative tyrosine motif ([FX]NPXY[FX]), as seen in Kir1.1, are also endocytosed through the CME pathway [11, 21, 35]. Here, recruitment into clathrin-coated vesicles appears to be independent of AP-2 but depends on CLASP proteins which either contain a phosphotyrosine-binding (PTB) domain and associate with either of PTB proteins, Disabled-2 (Dab2) and the autosomal recessive hypercholesterolemia (ARH) protein which localise to clathrin-coated structures [57, 79, 80]. [FX]NPXY[FX] motifs are also recognised by sorting nexin (SNX) proteins, endocytic proteins which contain phox-homology (PX) domains selective for endosomal phosphatidylinositol 3-phosphate (PtdIns(3)P) and function in cargo internalisation and endosomal sorting [19, 111, 116].

## Clathrin-independent endocytosis

While CME is recognised by its distinctive cytoplasmic coat and well-defined mechanism for selection of cell surface cargo, evidence supports the existence of additional endocytic routes with less distinctive pathways and cargo recruitment. Together these pathways are grouped by their lack of dependence on a clathrin coat and machinery and are termed clathrin-independent endocytosis (CIE). As these pathways have less distinct coated vesicles and adaptor proteins, to date, few cargo motifs have been exclusively associated with a single CIE pathway [45, 70]. It is currently unclear how many CIE pathways exist and if incorporation into these pathways is via recruitment into vesicles or bulk release from plasma membrane [107]. Three general regulators of these pathways have been defined. CIE pathways are divided into those regulated by small GTPases ARF6, Cdc42/ARF1 and Rho A [29]. Most CIE pathways are dependent on actin and sensitive to plasma membrane cholesterol concentration [1, 45, 82, 107]. Cholesterol levels play an important regulatory role in the function of small GTPase Cdc42, as well as the recruitment of cholesterol interacting membrane proteins (e.g. flotillins) which enhance some CIE pathways [6, 42, 82]. While CIE is often described as a non-selective process, internal sorting of CIE cargo is evident. ARF6 activity regulates post-endocytic trafficking of CIE cargo [34]. By activating phosphatidylinositol 4-phosphate 5-kinase ARF6 can increase phosphatidylinositol 4,5 bisphosphate (PIP2). Inactivation of ARF6 following internalisation results in reduced PIP2 levels and supports the fusion of endocytic vesicles with early endosomes (containing CME cargo) [51, 55, 61]. ARF6 activation is also required for recycling of CIE cargo usually from the recycling tubular endosomes back to the plasma membrane through activation of phospholipase D [55, 101]. Indeed, ion channels containing acidic cluster (termed potassium acidic clusters or KAC) have been shown to be recruited into an ARF6-regulated recycling pathway [44]. Addition of KAC to cargo enables its rapid recycling to the plasma membrane via a route which bypasses the early endosome. ARF6, Rac, Ras or Sfc activation has also been implicated in plasma membrane ruffling and macropinocytosis [60, 97, 120].

Until recently, caveolae were believed to represent a distinct CIE pathway and were proposed to regulate ion channel function by controlling their trafficking [5, 91]. Recent data demonstrate that caveolae are static at the plasma membrane and caveosomes remain attached to the cell surface [39, 89]. These developments suggest that caveolae may function in organising ion channel macrodomains; however, they appear not to enable channel endocytosis [39].

While it remains unclear whether cargo is actively selected at the plasma membrane or is released from retention to enable internalisation via CIE, evidence suggests that endocytosis of CIE cargo may occur by different pathways under different cellular conditions [3, 110].

## ARF6-dependent endocytosis is a key regulator of K_2P_1.1 (TWIK-1) surface expression

The wild-type protein of the first mammalian member of the K_2P_ channel family, K_2P_1.1 (two-pore domain weak-inwardly rectifying potassium channel or TWIK) achieves low levels of functional expression [65–67, 83]. Various explanations for this low channel function have been proposed including channel inhibition by SUMOylation or more recently the identification of a hydrophobic gate within the channel inner cavity which disrupts K^+^ flux [4, 37, 98]. Channel removal from the cell surface will also negatively impact channel function and increasing evidence suggests that independently of biophysical properties of this channel; K_2P_1.1 shows low levels of surface expression with a high level of regulation of its expression on the cell surface. Wang et al. provide evidence of K_2P_1.1 expression within astrocytes but demonstrate that these channels are predominantly located within the cytoplasm [117]. In 2004, Decressac et al. examined subcellular localisation of K_2P_1.1 in both adult mouse kidney and Madin-Darby canine kidney (MDCK) cells stably expressing the channel [26]. K_2P_1.1 localised to a subapical cellular domain in the kidney and polarised MDCK cells, which was confirmed to be an apical recycling compartment by co-localisation with endobrevin (vesicle associate membrane protein or Vamp-8) a marker for recycling endosomes and the perinuclear and vesiculotubular compartments. In non-polarised cells, K_2P_1.1 localised to the equivalent perinuclear recycling compartment. A di-isoleucine motif in the c-terminus of K_2P_1.1 (I293,294) was demonstrated to be instrumental in channel internalisation. Mutation of I293 results in increased channel surface expression and current [36]. The motif either enhances delivery or enables internalisation of the channel. Evidence suggests that it is the latter, as pulse chase experiments show nascent wild-type channels with intact di-isoleucine motifs being delivered to the cell surface and then rapidly (<10 min) recycled to subapical compartment [36]. Channels with mutated motifs remained on the cell surface. Investigation into the nature of the motif found that it operates in a manner comparable to the di-leucine motif ([DE]XXXL[LI]) which enables cargo endocytosis via the CME with the potential to be recognised by AP-1, AP-2 or AP-3 [114]. Interestingly, Feliciangeli et al. demonstrate that endocytosis enabled by the di-isoleucine in the c-terminus of K_2P_1.1 is sensitive to the activity of the mechanochemical GTPase, dynamin, required for vesicle scission in CME [36]. However, a clear role for ARF6-dependent endocytosis is also reported [26]. Elegant experiments from the same group using active and inactive forms of the small G protein ARF6 demonstrate that ARF6 exchange factor EFA6 interacts with an overlapping region of K_2P_1.1 in an ARF6 dependent manner [26]. Dominant negative ARF6 was shown to promote surface expression of K_2P_1.1 [26, 59].

These findings suggest an interplay between the CME and CIE pathways with ARF6 enhancing CME [25, 61, 88]. D’Souza-Schorey et al. first reported this in 1995 when they observed that a constitutively active ARF6 mutant (ARF6Q67L) inhibited transferrin receptor internalisation while the inactive ARF6 (ARF6T27N) prevented its recycling to the plasma membrane [25]. A clear link between CME pathways and ARF6 is apparent. Indeed, an ARF6 GTPase activating protein (GAP), SMAP1, which converts ARF6 from GTP bound to an inactive GDP bound state, directly binds clathrin. Additionally ARF6-GTP has been proposed as a source of GTP for dynamin-dependent scission of clathrin buds [88]. Active ARF6 may also enable recruitment of AP2 to the plasma membrane due to its role in PIP2 production which could then recruit K_2P_1.1 to clathrin coated pits via it c-terminal di-isoleucine motif [51, 61].

The mode of interplay between the CME pathway and ARF6 for K_2P_1.1 sorting has yet to be fully ascertained; however, it is clear that K_2P_1.1 is internalised to a subapical compartment via di-isoleucine motif and is recycled in an ARF6-dependent manner, with the consequence of K_2P_1.1 being sequestered below the cell surface [26, 36]. This cellular strategy would explain the maintenance of a low level of this leak channel on the cell surface that could then be rapidly delivered to the plasma membrane or transported onto the endocytic pathway (early endosomes) in response to external cues. Indeed, activation of the serotonergic receptor results in increased K_2P_1.1 surface expression in the wild-type protein but not when the di-isoleucine motif has been disrupted [36]. 5-HT has previously been demonstrated to inhibit entorhinal cortex neurons in a K_2P_1.1-dependent manner [27]. These data provide insight into this mechanism and suggest that 5-HT triggers the release of K_2P_1.1 from the subapical recycling compartment increasing its cell surface expression and resulting in neuronal depolarisation. The molecular mechanisms of many aspects of these regulatory processes are still lacking significant detail but a clear role of endocytosis in the regulation of K_2P_1.1 is undeniable.

## Does bulk endocytosis control surface expression of K_2P_18.1 (TRESK)?

An alternative strategy for neuronal cells to control the surface expression of potassium leak channels is proposed for K_2P_18.1 (also TWIK-related spinal cord potassium channel or TRESK). K_2P_18.1 exhibits predominantly neuronal expression and disruption of its function has been implicated in playing a role in migraine with aura [33, 63, 108]. TRESK contains a consensus site for the calcium-dependent phosphatase, calcineurin, within its large cytosolic loop between transmembrane domains 2 and 3 [24]. Calcineurin plays a role in activity-dependent bulk endocytosis (ADBE) in nerve termini [119]. While no direct experimental evidence has been provided for TRESK internalisation via ADBE in activated nerve termini, its recruitment to the complex and the indiscriminate nature of this endocytic pathway certainly supports the concept.

## K_2P_2.1 binding partners impact channel plasma membrane density

Following K_2P_2.1’s (TWIK-related potassium channel 1 or TREK-1) molecular and biophysical characterisation, it rapidly became recognised as an important regulator of neuronal function [84]. K_2P_1.1 is implicated in depression, polymodal pain perception, diseases related to blood-brain barrier dysfunction and anaesthesia [2, 9, 38, 48, 52, 56]. Due to the neurological physiology and pathophysiology it impacts, modes of long-term regulation of K_2P_2.1 are particularly sought after. A-kinase anchoring protein (AKAP150), microtubule-associated protein (Mtap2) and neurotensin receptor 3 (NTSR3 also known as sortilin) have been identified as binding partners to K_2P_2.1 [76, 105, 106]. Both Mtap2 and NTSR3 are proposed to act by altering channel density. While Mtap2 interaction with K_2P_2.1 was shown to enhance channel density, it is unclear if this is due to enhanced delivery or reduced recovery at the plasma membrane [105]. NTSR3 promotes endocytosis and lysosomal sorting of neuronal cargo [18, 115, 118]. Interaction of a partial propeptide of NTSR3 (referred to as Spadin) with K_2P_2.1 resulted in 80 % of the complex being observed within the cell [76]. The researchers propose that activation of NTSR3 (through application of Spadin) results in endocytosis of both receptor and channel with likely targeting to TGN or lysosomes. To date, only protein interactions and channel density reduction have been confirmed experimentally and the precise pathways and channel destination have yet to be determined.

## K_2P_3.1 utilises both CME and CIE pathways

K_2P_3.1 (TWIK-related acid sensitive potassium channel 1 or TASK-1) and K_2P_9.1 (TWIK-related acid sensitive potassium channel 3 or TASK-3) show a high degree of homology in sequence and biophysical properties with both channels sensitive to external pH and regulated by similar modulators [8, 31, 58, 99]. These channels have been proposed to function as heterodimers in some cells and indeed the forward transport of both channels appears to undergo similar regulation through post-translational modification and binding partner recruitment [23, 41, 71, 72, 85, 86, 100, 103, 121]. However, when considering K_2P_3.1 and K_2P_9.1 internalisation and subsequent sorting, these channels appear to diverge in their regulation. As the c-termini of these channels have a critical role in channel sorting and the c-termini of K_2P_3.1 and K_2P_9.1 show only 34 % homology, this divergence is not wholly unexpected.

Under unstimulated conditions, both K_2P_3.1 and K_2P_9.1 are internalised and appear within the early endosome within minutes of permitting endocytosis (through removal of temperature block) [71]. Quantification of the number of size-defined vesicles containing either of the internalised channels enabled comparison of the transit of both channels through the endocytic system. At specific time points, a higher number (>50 % increase) of endocytosed vesicles containing K_2P_9.1 compared to K_2P_3.1 were consistently observed [71]. This suggests that under unstimulated conditions either K_2P_9.1 is endocytosed more readily than K_2P_3.1 or indeed that it is retained within the endocytic pathway for longer (i.e. not shuttled back to the membrane or degraded). Both channels were found to colocalise with clathrin, and the number of endocytosed vesicles positive for both clathrin and either of the channels was dramatically reduced (∼50 % reduction) following disruption of the CME pathway by dynasore (a powerful dynamin GTPase inhibitor which prevents fission of clathrin buds) [40, 71, 74, 102]. Continued channel internalisation following inhibition of clathrin bud fission suggests the possibility of these channels also utilising an alternate or clathrin independent pathway for internalisation [71]. Fractionation experiments performed by Inoue et al. support this view as they observed the majority of K_2P_9.1 within a cell fraction in which transferrin receptor (a known cargo for CME) was also observed, but while K_2P_3.1 was also observed within this fraction, it was also observed in a fraction containing flotillin which is suggestive of CIE [74]. Endocytosed channels were observed in both the recycling (Rab 11 positive vesicles) and degradative (Lamp1 positive) pathways in a number of studies using cells with either endogenous or heterologous channel expression [40, 71, 74, 102].

Stimulated K_2P_3.1 internalisation has been demonstrated in a number of systems and is proposed to utilise specific motifs within the channel c-terminus. Nerve growth factor (NGF) activation of tyrosine kinase, TrKA, has been proposed to induce CME of K_2P_3.1 (but not K_2P_9.1) [74]. NGF treatment of either acutely isolated rat adrenal medulla cells which express K_2P_3.1 channels or a rat adrenal medulla cell line from a pheochromocytoma (PC12 cells) which express both K_2P_3.1 and K_2P_9.1 resulted in decreased functional expression of K_2P_3.1 and increased cytosolic expression of the channel [74]. Matsuoka et al. propose NGF stimulates K_2P_3.1 internalisation through CME. However, the possibility that some of the observed effects following NGF treatment (decreased functional expression and increased cytosolic localisation of the channel) are due to disruption of channel forward transport is not completely ruled out. A di-leucine motif (LL263/264) within K_2P_3.1 c-terminus has been implicated in recruiting the channel to clathrin pits, while two c-terminal potential tyrosine motifs (YAEM or YSIP; Y317 and Y340 in rat K_2P_3.1) were found not to contribute to the effect [74].

While Y317 was found not to be essential to NGF induced channel internalisation, a separate study found this motif to be critical to K_2P_3.1 endocytosis in the human ortholog. Reningunta et al. demonstrate a direct interaction between a member of the Q-SNARE family (Q-soluble *N*-ethylmaleimide-sensitive factor attachment protein receptor) syntaxin 8 (stx8) and K_2P_3.1 and utilising recombinant expression systems demonstrate that stx8 suppresses K_2P_3.1 current and surface expression [102]. This interaction was not observed with either stx7 (another member of the SNARE family) or K_2P_9.1. SNAREs operate by using a combination of various members of the Q and R-SNAREs to enable organelle-specific docking and fusion [50, 112]. Regulation of K_2P_3.1 surface expression by stx8 is lost by ablating a region of interaction on stx8 or by disrupting CME. Disruption of the Y317 (critical to YAEV motif in human K_2P_3.1 but absent from K_2P_9.1) disabled stx8-associated internalisation of K_2P_3.1 [102]. This work further demonstrated that both stx8 and K_2P_3.1 are endocytosed in a cooperative manner and within the same vesicles. An attractive hypothesis proposed by the authors is that cooperative recruitment of K_2P_3.1 and stx8 may influence their final destination through interaction with SNARE proteins localised to distinct subcellular compartments.

Protein kinase C (PKC) activation results in a decrease in K_2P_3.1 current and cell surface expression [7, 40]. Human embryonic kidney (HEK293T) cells heterologously expressing K_2P_3.1 or rat cerebellar granule neuron (CGN) with endogenous expression of the channel treated with phorbol myristate acetate (PMA), a potent activator of PKC resulted in K_2P_3.1 but not K_2P_9.1 internalisation and localisation within distinct intracellular puncta [40]. Similarly, activation of mGluR had a comparable effect with reduced current and increased K_2P_3.1 internalisation through CME. An REK motif analogous to a PKC-endocytosis motif identified and characterised in the dopamine transporter was found to be central to PKC-mediated endocytosis of K_2P_3.1 but lacking in K_2P_9.1 [40, 49, 68]. Gabriel et al. propose that this motif (SRERKLQYSIP) is similar to a mode II 14-3-3 binding motif and present evidence supporting the hypothesis that PKC-mediated endocytosis requires 14-3-3 [40]. The rationale for targeting 14-3-3β alone or the identification of the requisite phosphorylation site were lacking but intriguingly 14-3-3β knockdown had a negative impact on both channel cell surface expression and current following PKC activation. These findings present interesting avenues to explore in terms of the role played by 14-3-3 in balancing K_2P_3.1 forward transport and retrieval from the cell surface.

## How much can signaling motifs tell us?

Endocytic signaling motifs are not conserved sequences but rather degenerate motifs in which two or three residues are critical for signal recognition. Recruitment to specific pathways is influenced by motif sequence; however, the ultimate fate of cargo following internalisation (degradation or recycling) is often determined not only by the pathway into which the protein is recruited but also by the affinity of adaptor proteins for variations within motif sequences [3, 110]. YXXϕ motifs, for example are recognised by AP proteins and are essential for the rapid internalisation of cargo from the plasma membrane [10, 114]. However, their function is not limited to endocytosis, since the same motif is implicated in the targeting of transmembrane proteins to lysosomes and lysosome-related organelles [10]. Evidence suggests that the variable residues (XXϕ) within the motif together with the phosphorylation state of the motif are central to recruiting specific AP isoforms and hence cargo destination. For example, YXXϕ signals involved in lysosomal targeting customarily have a Gly preceding the critical Tyr, while acidic X residues result in lysosomal sorting [10, 14, 114]. This scenario is made all the more complicated by many transmembrane proteins containing more than one motif (Table 1) and the functionality of the motif being influenced by its position within the channel cytosolic domain and flanking residues. The impact of post-translational modifications within the motif and surrounding residues (ubiquitination, palmitoylation, phosphorylation, glycosylation) have also been well documented to impact motif usage and cargo fate [17, 20, 77, 94]. When examining the K_2P_ family of channels for prominent endocytic motifs, the majority of channels displayed an array of putative endocytic and sorting motifs (Table 1). Interestingly, none of the K_2P_ channels contained the tyrosine motif [FX]NPXY[FX], previously identified in other ion channels [35]. Many K_2P_ channels displayed multiple putative Yxxϕ motifs with up to four in some channels (Table 1). Many other membrane proteins contain a number of tyrosine motifs which on experimental analysis are not functionally relevant or may only be utilised under specific environmental conditions [17, 110]. Di-leucine or di-isoleucine motifs were only identified in K_2P_1.1 and K_2P_3.1 and both of these have been demonstrated to be functionally significant [36, 74]. Putative short proline-rich domains, which are proposed to enable sorting between endosomes and the TGN, were identified in five K_2P_ channels, and for K_2P_15.1 (TASK-5) and K_2P_16.1 (TALK-1) these were the only putative endocytic sorting motifs identified (Table 1). While putative short acidic clusters (KAC) which are associated with protein retention within ARF6 positive recycling compartments were found in eight of the 14 human K_2P_ channels examined [44]. As only a handful of the predicted motifs have been experimentally determined, and motif usage depends on both protein and cellular context, functional motifs and the fate of channels due to possession of a signal motif will need to be explored experimentally.

**Table 1:** Putative endocytic and sorting signaling motifs identified through in silico analysis of human K_2P_ family members. Residue number of critical or first residue within the human motif provided. Residues critical to motif recognition are in bold

	**Y**xx**ϕ**	**DE**xxx**L[LI]**	**KAC**	**Pro-rich**	**L**ϕxϕ**[DE]**
**K** **2P** **1.1 (TWIK-1)**	**Y** 167FH**L**	**D**QVH**I** 293 **I**	**D** 287 **KDED**		**L** 261VVL**E**
**K** **2P** **2.1 (TREK-1)**	**Y** 254FV**V**				
	**Y** 277LD**F**				
	**Y** 281KP**V**				
	**Y** 348DK**F**				
**K** **2P** **3.1 (TASK-1)**	**Y** 138LL**H**	**E**HRA**L** 263 **L**	**E** 252 **DEKRD**		
	**Y** 300AE**V**				
	**Y** 323SI**P**				
	**Y** 353SD**T**				
**K** **2P** **4.1 (TRAAK)**				**P** 306 **PPPCP**	**L** 344aFI**D**
**K** **2P** **5.1 (TREK-2)**	**Y** 301ND**L**				**L** 413IFQ**D**
	**Y** 410HP**L**				**L** 429SDE**E**
	**Y** 483EQ**L**				**L** 443AGE**E**
					**L** 459NMG**E**
**K** **2P** **6.1 (TWIK-2)**	**Y** 308AS**L**		**D** 282 **EDDRVD**	**P** 272 **PPCP**	
**K** **2P** **9.1 (TASK-3)**			**E** 252 **DERRDAEE**		
**K** **2P** **10.1 (TREK-2)**	**Y** 478KT**F**		**D** 482 **EEKKEEE**		
**K** **2P** **12.1 (THIK-2)**			**D** 356 **SDAE**	**P** 6 **RPPP**	
**K** **2P** **13.1 (THIK-1)**			**E** 335 **SDTD**		**L** 291RKM**D**
**K** **2P** **15.1 (TASK-5)**				**P** 261 **SPRPP**	
**K** **2P** **16.1 (TALK-1)**				**P** 301 **LPLP**	
**K** **2P** **17.1 (TASK-4)**	**Y** 236 **PLW**		**D** 295 **REPE**		
	**Y** 241 **KNM**				
**K** **2P** **18.1 (TRESK)**	**Y** 123PV**T**				
	**Y** 315FC**F**				
	**Y** 343II**V**				
	**Y** 365KN**V**				

Even with our limited understanding of the endocytic processes utilised to regulate K_2P_ channel functional expression, it is clear how versatile and important endocytosis could be in controlling the cell surface expression of such physiologically relevant proteins. As these channels enable K leakage from cells when they are expressed on the cell surface, their regulated removal from the plasma membrane and retention within recycling endosomes close to the cell surface appears a shrewd strategy to rapidly alter channel density in response to environmental stimuli. Equally, sorting these channels for destruction to regulate the optimal cell surface density either under unstimulated or stimulated conditions is a mode of regulation which if molecularly defined could have significant physiological and clinical implications.
